# Integrated transcriptomic and proteomic analysis of the molecular cargo of extracellular vesicles derived from porcine adipose tissue-derived mesenchymal stem cells

**DOI:** 10.1371/journal.pone.0174303

**Published:** 2017-03-23

**Authors:** Alfonso Eirin, Xiang-Yang Zhu, Amrutesh S. Puranik, John R. Woollard, Hui Tang, Surendra Dasari, Amir Lerman, Andre J. van Wijnen, Lilach O. Lerman

**Affiliations:** 1 Division of Nephrology and Hypertension, Mayo Clinic, Rochester, Minnesota, United States of America; 2 Department of Health Sciences Research, Mayo Clinic, Rochester, Minnesota, United States of America; 3 Department of Cardiovascular Diseases, Mayo Clinic, Rochester, Minnesota, United States of America; 4 Department of Orthopedic Surgery, Mayo Clinic, Rochester, Minnesota, United States of America; Center for Molecular Biotechnology, ITALY

## Abstract

**Background:**

Mesenchymal stromal/stem cell (MSC) transplantation is a promising therapy for tissue regeneration. Extracellular vesicles (EVs) released by MSCs act as their paracrine effectors by delivering proteins and genetic material to recipient cells. To assess how their cargo mediates biological processes that drive their therapeutic effects, we integrated miRNA, mRNA, and protein expression data of EVs from porcine adipose tissue-derived MSCs.

**Methods:**

Simultaneous expression profiles of miRNAs, mRNAs, and proteins were obtained by high-throughput sequencing and LC-MS/MS proteomic analysis in porcine MSCs and their daughter EVs (n = 3 each). TargetScan and ComiR were used to predict miRNA target genes. Functional annotation analysis was performed using DAVID 6.7 database to rank primary gene ontology categories for the enriched mRNAs, miRNA target genes, and proteins. STRING was used to predict associations between mRNA and miRNA target genes.

**Results:**

Differential expression analysis revealed 4 miRNAs, 255 mRNAs, and 277 proteins enriched in EVs versus MSCs (fold change >2, p<0.05). EV-enriched miRNAs target transcription factors (TFs) and EV-enriched mRNAs encode TFs, but TF proteins are not enriched in EVs. Rather, EVs are enriched for proteins that support extracellular matrix remodeling, blood coagulation, inflammation, and angiogenesis.

**Conclusions:**

Porcine MSC-derived EVs contain a genetic cargo of miRNAs and mRNAs that collectively control TF activity in EVs and recipient cells, as well as proteins capable of modulating cellular pathways linked to tissue repair. These properties provide the fundamental basis for considering therapeutic use of EVs in tissue regeneration.

## Introduction

Mesenchymal stromal/stem cells (MSCs) are being tested in clinical trials to evaluate their therapeutic efficacy in a broad spectrum of diseases [1]. The potential medical benefits of MSCs reside in their remarkable differentiation capabilities, as well as potent pro-angiogenic and immunomodulatory properties [2]. Given that MSCs can be easily obtained from a variety of tissues, including adipose tissue, these cells offer an important advantage in clinical applications compared to other stem cell types.

Accumulating evidence indicate that the reparative effects of MSCs are primarily paracrine-mediated, including secretion of extracellular vesicles (EVs) composed of microvesicles and exosomes that mediate intercellular communications. Microvesicles (50-1000nm) are formed by outward budding and fission of the plasma membrane, whereas exosomes (40-100nm) are released as a consequence of multi-vesicular endosome fusion with the plasma membrane. Studies have shown that MSCs produce the highest amount of EVs among different cell types [3]. Furthermore, MSC-derived EVs recapitulate the pro-angiogenic and immunomodulatory functions of their parent MSCs by acting as vehicles for transferring genes, micro-RNA (miRNAs), and proteins to recipient cells [4, 5]. Consequently, EVs might constitute a promising platform for non-cellular regenerative therapies to complement the use of MSCs in tissue regeneration and repair, which are currently being tested in several clinical trials that evaluate the safety, tolerability, and efficacy of MSCs in treating a myriad of diseases [6].

Consistent with these concepts, we have previously shown that porcine adipose tissue-derived MSCs contain a combination of mRNAs and miRNAs that are in principle capable of regulating the expression of genes that control angiogenesis, adipogenesis, and other pathways in targeted cells [7]. We have also recently shown that these EVs contain proteins that may contribute to molecular mechanisms linked to MSC-mediated tissue repair, including modulators of angiogenesis, blood coagulation, extracellular matrix remodeling, apoptosis, and inflammation [8]. However, the regulatory interactions among components of the MSC regulome remain poorly characterized. Understanding the interactions among the different components of the EV regulome is critical for understanding the mechanisms responsible for MSC-induced tissue repair and ensuring the clinical success of MSC therapy.

In the current study, we performed a comprehensive integrated analysis of the mRNA and miRNA transcriptomes and proteome of porcine MSC-derived EVs, to assess whether these different types of molecular cargo are capable of mutual regulation, are functionally interrelated, and/or may together target distinct cellular pathways that converge on the same biological goals. Our results indicate that porcine MSC-derived EVs are selectively enriched for mRNAs and miRNAs that are predicted to interact and control the activity of transcription factors, while EV proteins are capable of modulating multiple cellular phosphorylation pathways. Collectively, these observations support the premise that EVs selectively incorporate genetic material and regulatory proteins that support MSC-mediated tissue regeneration.

## Materials and methods

**Fig 1:**
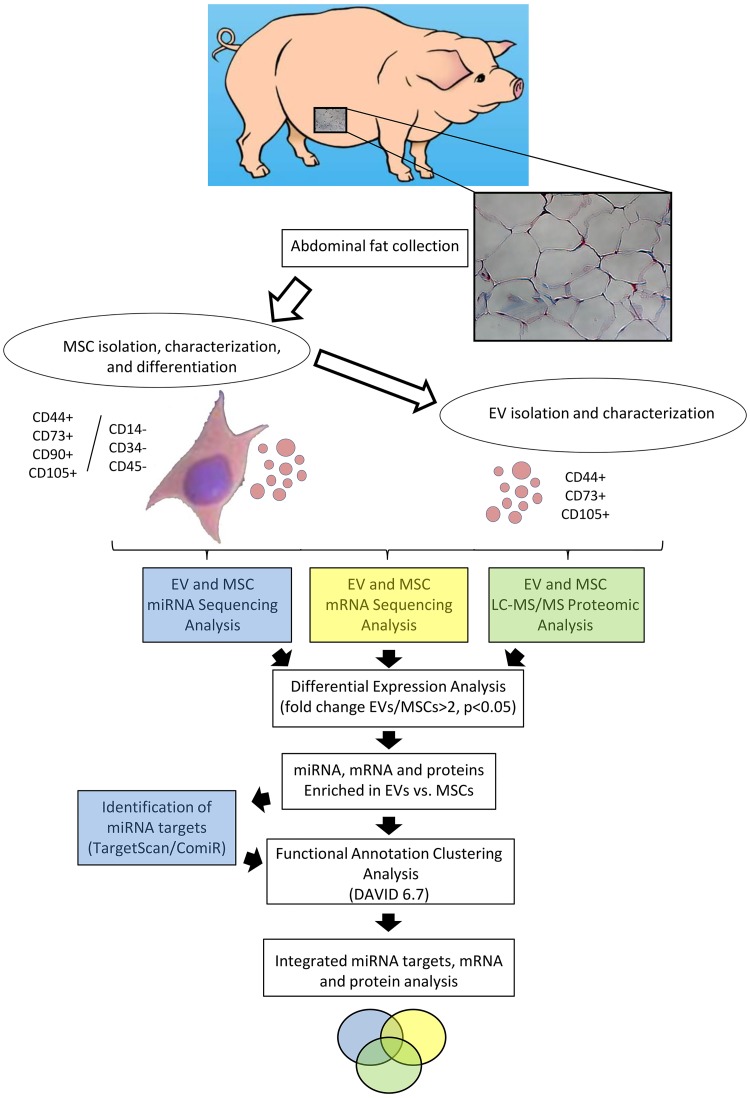
Overview of experimental design and data analysis. Abdominal fat was collected from female domestic pigs, and mesenchymal stem cells (MSCs) and their daughter extracellular vesicles (EVs) isolated and characterized. mRNA and miRNA sequencing analysis and LC-MS/MS proteomic analysis were performed in both MSCs and EVs (n = 3 each). Differentially expression analysis was performed and EV-enriched miRNA, mRNA, and proteins identified. miRNA predicted targets were identified with TargetScan and ComiR. Functional annotation clustering analysis was performed using DAVID 6.7 database to obtain a ranking of primary gene ontology categories for the enriched mRNA, miRNA target genes, and proteins. Venn diagrams were used to visualize genes shared between each group and their interactions, and STRING to predict associations between mRNA and miRNA target genes.

The study was approved by the Mayo Clinic Animal Care and Use Committee. Fig 1 summarizes the experimental design and data analysis. Abdominal fat was collected from 3 female domestic pigs. Animals were anaesthetized with 0.25g of intramuscular tiletamine hydrochloride/zolazepam hydrochloride and 0.5g of xylazine, and anesthesia was maintained with intravenous ketamine (0.2mg/kg/min) and xylazine (0.03mg/kg/min). The abdominal region lateral to the umbilicus was draped and prepped under standard sterile technique using alcohol and local anesthesia (2mL 2% lidocaine). A small superficial 0.5cm skin incision was performed, and adipose tissue collected and stored in sterilized tubes. Analgesics (topical 1% lidocaine; Buprenorphine 0.005–0.02mg/kg IM, IV q6-12h) were administered for 24hrs days after fat biopsy.

### MSC and EV isolation, characterization, and culture

Primary MSCs were isolated from abdominal fat (5-10g) using collagenase-H, cultured for 3 weeks in advanced MEM medium (Gibco/Invitrogen) supplemented with platelet lysate (PLTmax, Mill Creek Life Sciences, Rochester, MN), and used at the third passage, as previously shown [7, 9–13]. MSC cultures did not include any animal products with the exception of porcine MSCs. Cells were characterized in vitro by positive immuno-fluorescent staining and fluorescence activated cell sorting analysis for CD44, CD73, CD90, and CD105, as previously described [9, 10, 14] and consistent with our observations in human MSCs [15, 16].

EVs were isolated from MSC supernatants (10x10^6^ cells) by ultracentrifugation, as previously described [7]. Samples were centrifuged at 2,000g for 20min, the supernatant collected, and samples subsequently centrifuged at 100,000g for 1h at 4°C. EVs were then collected, suspended in wash buffer medium 199, and centrifuged at 100,00g for 1hr. Transmission electron microscopy was performed to investigate size and structure of MSC-derived EVs using digital electron microscopy (JEOL 1200 EXII, Mayo Clinic’s electron microscopy core) [8]. EVs were then characterized based on the expression of both EV (CD9, CD29, and CD63) and MSC (CD73 and CD105) surface markers by western blot (AbD Serotec cat#: MCA1189 and AA120-175, and Abcam cat#: ab61873, ab115289, and ab135528). In addition, EV concentration and size distribution were assessed by nanoparticle tracking analysis (NTA) using NanoSight NS300. EVs were diluted with PBS and samples continuously run through a flow-cell top-plate at 25μL/min. Three videos (120 seconds each) of Brownian motion of nanoparticles were recorded and 1,000 completed tracks analyzed using NTA 2.3.5.

### Transcriptome analysis

RNA sequencing analysis was performed as described [16]. Sequencing RNA libraries were prepared according to the manufacturer’s protocol (TruSeq RNA Sample Prep Kit v2, Illumina) and loaded onto flow cells (8-10pM) to generate cluster densities of 700,000/mm^2^ following the standard protocol for the Illumina cBot and cBot Paired-end cluster kit version-3. Cells were sequenced on an Illumina HiSeq 2000 using TruSeq SBS kit version 3 and HCS v2.0.12 data collection software and data analyzed using the MAPRSeq v.1.2.1 system and the Bioinformatics Core standard tool, which includes alignment with TopHat 2.0.6 [17, 18] and gene counts with the featureCounts software [19]. miRNA-Seq data were analyzed using CAP-miRSeq v1.1 [20] and normalization and differential expression analysis performed using edgeR 2.6.2 [21]. Gene expression was normalized to 1 million reads and corrected for gene length (reads per kilobasepair per million mapped reads, RPKM), and miRNA expression levels expressed as normalized total reads.

### Proteome analysis

Liquid chromatography mass spectrometry (LC-MS/MS) proteomic analysis was performed as previously described [22, 23]. MSC and EV pellets were solubilized and lysed, and protein samples denatured by incubation at 85°C for 10min. Aliquots were resolubilized in reducing sample buffer and samples electrophoresed in 4–20% TGX Ready gels at 200V for 30min. Gel sections were digested with trypsin [23], and peptides extracted and transferred onto a 35cmx100μm PicoFrit column 9 (NewObjective), self-packed with Agilent Poroshell 120S 2.7μm EC-C18 stationary phase, using a Dionex UltiMate 3000 RSLC LC system (Thermo-Fisher Scientific). Peptides were separated and eluting peptides analyzed using a QExactive mass spectrometer (Thermo-Fisher Scientific). Label-free peptide MS1 intensity-based methods were used to identify differentially expressed proteins between MSCs and EVs. Data quality was assessed using MaxQuant 1.5.1) software [24] and reversed protein sequences appended to the database for estimating protein identification false discovery rates (FDRs). Protein group intensities of each sample were log_2_ transformed, normalized, and modeled using a Gaussian-linked generalized linear model. Data was normalized by protein loading, and differential p-values FDR-corrected using the Benjamini-Hochberg-Yekutieli procedure [25].

### Validation of RNAseq and proteomic analysis

SMAD2 and POU2F1 mRNAs, miR-140-3p and miR-378 miRNAs, and C2 and TGFβ-1 proteins, which were all enriched in EVs, were selected for validation, and their expression in EVs and MSCs measured by quantitative PCR and Western blot, respectively.

### Human Umbilical Vein Endothelial Cells (HUVEC) studies

In vitro experiments were performed in HUVECs (Cell Applications, cat# 200K-05f). Cells were characterized by the expression of the endothelial markers CD31 and cultured and maintained at 37°C in endothelial culture media supplied by the company. Then, HUVECs were sub cultured in T-75 flasks either untreated or treated with red fluorescence labeled (PKH26, Sigma) EVs (100μg of protein each) for 24hrs. Expression of the candidate miRNAs (miR-140-3p and miR-378), genes (SMAD2 and POU2F1), and proteins (C2 and TGFβ-1) was measured by quantitative PCR and western blot (six wells per group).

### Integrated bioinformatic analysis of miRNA, mRNA, and protein data

miRNA, mRNA, and proteins with fold-change (EVs/MSCs) >2 and p values <0.05 (EVs vs. MSCs, 2-tail Student t-test) were considered enriched in EVs [16]. We used TargetScan 7.1 (http://www.targetscan.org/vert_71) and ComiR (http://lagavulin.ccbb.pitt.edu/comir/index.php) to predict target genes of miRNAs enriched in EVs. Proteins enriched in EVs were classified by their molecular function and cellular localization using Protein Analysis Through Evolutionary Relationships (PANTHER) [26]. Functional annotation clustering analysis was performed using DAVID6.7 database (http://david.abcc.ncifcrf.gov/) [27, 28] to obtain a ranking of primary gene ontology categories for the enriched mRNAs, miRNA target genes, and proteins. Three-way Venn diagrams were constructed using VENNY 2.1 (http://bioinfogp.cnb.csic.es/tools/venny/) to visualize common mRNAs, miRNA target genes, and proteins upregulated in EVs, as well as common TF mRNAs, miRNA target TFs, and TF proteins enriched in EVs. Functional annotation clustering analysis of shared genes was performed using DAVID6.7. Search Tool for the Retrieval of Interacting Genes (STRING) version 9.1 (http://string-db.org/) was used to predict associations between mRNA TFs and miRNA target TFs.

## Results

### MSC and EV characterization

**Fig 2:**
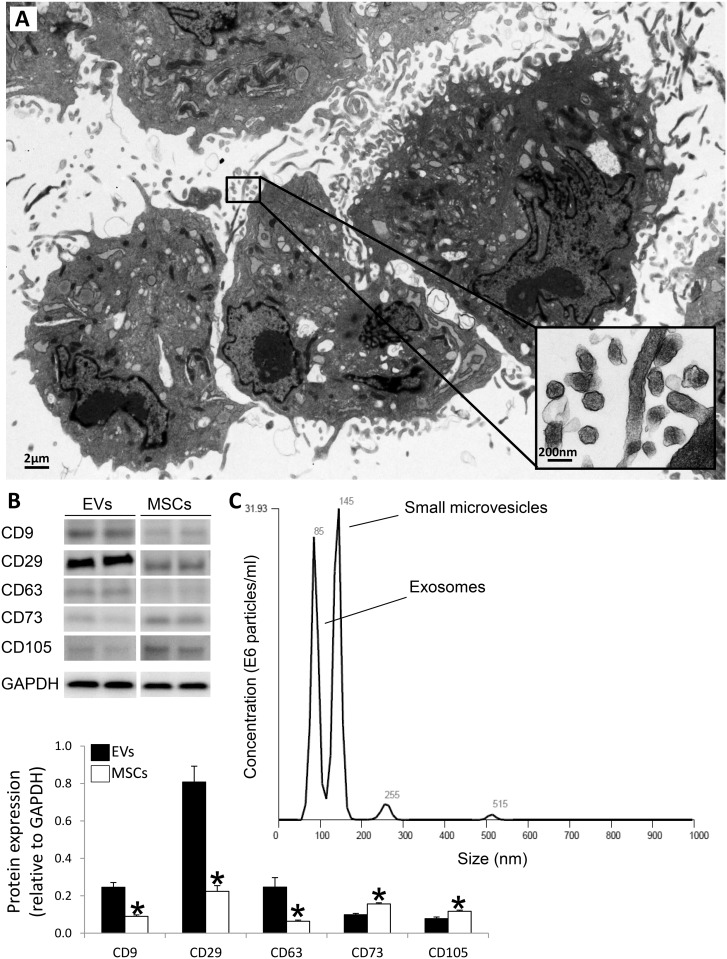
Characterization of MSC-derived EVs. A: Transmission electron microscopy showing cultured MSCs releasing EVs. B: EVs express common EV (CD9, CD29, and CD63) and MSC (CD73 and CD105) markers. C: Size distribution of isolated EVs revealed a similar proportion of small microvesicles and exosomes.

MSCs expressed mesenchymal markers (CD44, CD90, and CD105), were negative for endothelial (CD31) and inflammatory (CD14 and CD45) markers, and trans-differentiated into osteocytes, chondrocytes, and adipocytes in-vitro, as previously described [7, 9–13]. Transmission electron microscopy demonstrated that cultured MSCs release microparticles (Fig 2A) that express EV (CD9, CD29, and CD63) and MSC (CD73 and CD105) surface markers (Fig 2B). EV size/concentration distribution showed a similar proportion of exosomes and small microvesicles (Fig 2C). The prominent production of EVs by MSCs provides a compelling argument for the general working hypothesis that EVs are integral components of the basic tissue repair machinery of MSCs.

### 4 miRNAs were enriched in EVs

**Fig 3:**
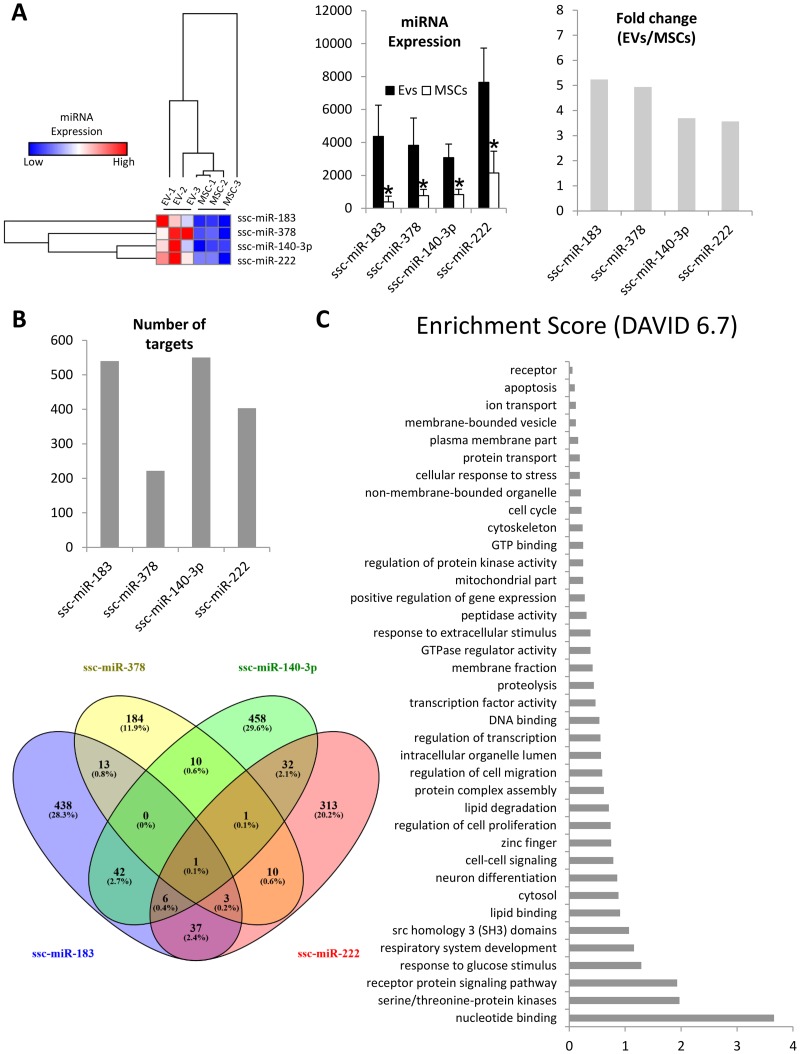
miRNA enriched in EVs. A. Heat map, miRNA expression, and fold change showing that ssc-miR-183, ssc-miR-378, ssc-miR-140-3p, and ssc-miR-222 were upregulated in EVs compared to MSCs. B. Number (top) and distribution (bottom) of miRNA targets of EV-enriched miRNAs. C: Functional annotation clustering of miRNA targets.

miRNA-Seq analysis identified a total of 413 miRNAs, among which miR-183, miR-378, miR-140-3p, and miR-222 were enriched in EVs compared to MSCs (Fig 3A). Target prediction analysis revealed that miR-183, miR-140-3p, and miR-222 target over 400 genes, whereas miR-378 targets only 222 genes (Fig 3B). Venn diagram analysis shows a small number of miRNAs sharing target genes (Fig 3B). Functional annotation clustering analysis of mRNAs targeted by this set of miRNAs identified genes primarily associated with transcription (Fig 3C), including SMAD Family Member 2 (SMAD2), POU Class-2 Homeobox 1 (POU2F1), MDM4, P53 Regulator (MDM4), and One Cut Homeobox 2 (ONECUT2). These observations suggest that the miRNA cargo of EVs may alter the phenotype of recipient cells by mediating translational control of transcription factors.

### 255 mRNAs were enriched in EVs

**Fig 4:**
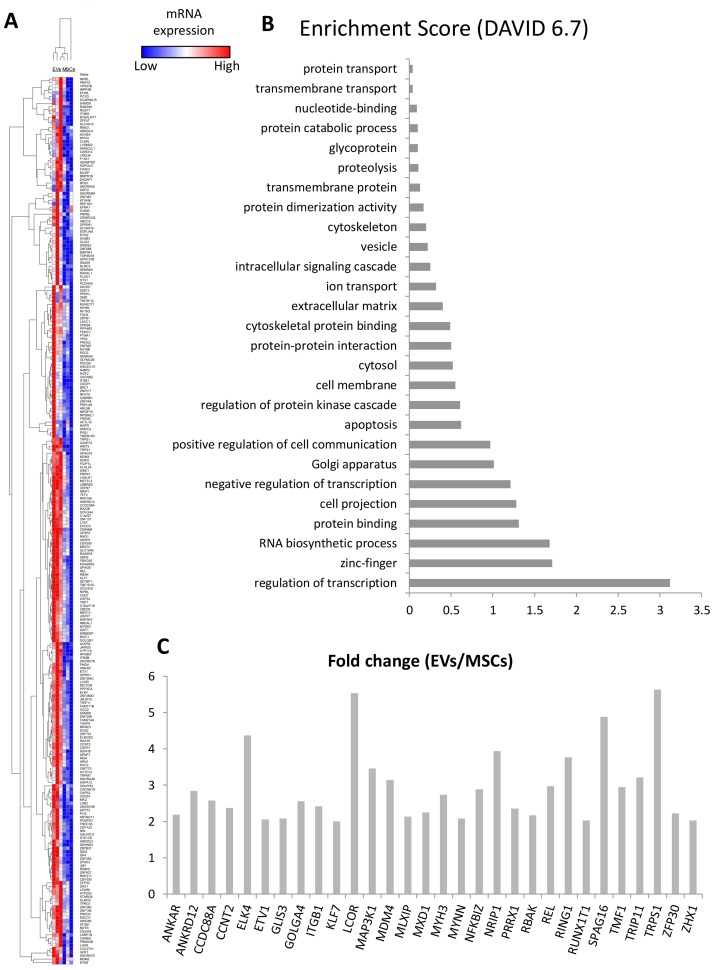
mRNA enriched in EVs. A. Heat map of 255 mRNAs enriched in EVs. B. Functional annotation clustering of EV-enriched mRNAs. C: Fold change (EVs/MSCs) of 30 genes involved in regulation of transcription enriched in EVs.

Of all annotated genes (n = 11,956), mapping of RNA reads revealed 255 mRNAs that were upregulated in EVs compared to their parent MSCs (Fig 4A). Annotation analysis showed that EVs selectively contain genes encoding positive or negative regulators of transcription (Fig 4B), including Transcriptional Repressor GATA Binding 1 (TRPS1), ELK4, ETS Transcription Factor (ELK4), Kruppel-Like Factor 7 (KLF7), and Nuclear receptor interacting protein 1 (NRIP1) (Fig 4C). The presence of TF mRNAs in EVs conforms to the model that EVs may provide genetic instructions to recipient cells.

### 277 proteins were enriched in EVs

**Fig 5:**
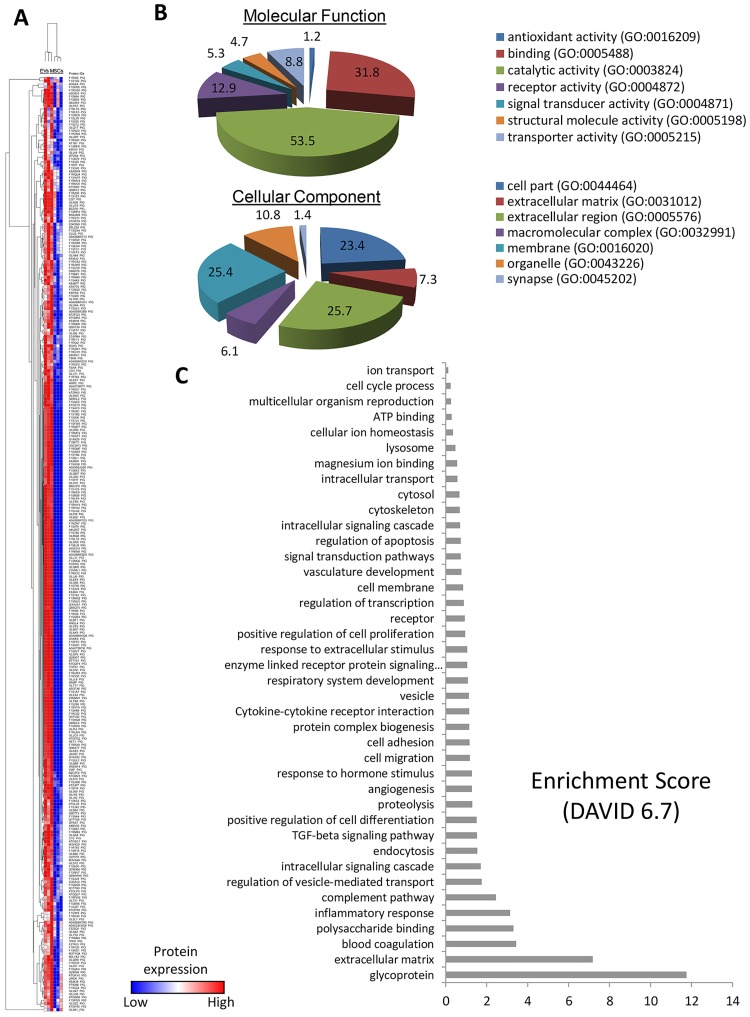
Proteins enriched in EVs. A. Heat map of 277 proteins upregulated in EVs compared to MSCs. B. Panther analysis of molecular function and cellular component of proteins upregulated in EVs. C. Functional annotation clustering of EV-enriched proteins.

Proteomic analysis identified a total of 5,623 proteins, including 277 selectively enriched in MSC-derived EVs (Fig 5A). The majority of the proteins enriched in EVs showed catalytic activity (53.5%), and were mostly distributed in the extracellular region (Fig 5B). Functional classification revealed an important diversity of biological roles, with glycoproteins and extracellular matrix proteins showing the highest enrichment scores, whereas the remaining categories, similarly distributed, included blood coagulation, inflammatory response, TGF-β signaling pathway, and angiogenenic proteins (Fig 5C). This proteomic cargo is consistent with biological activities of EVs in tissue repair.

### Validation of RNAseq and proteomic analysis

**Fig 6:**
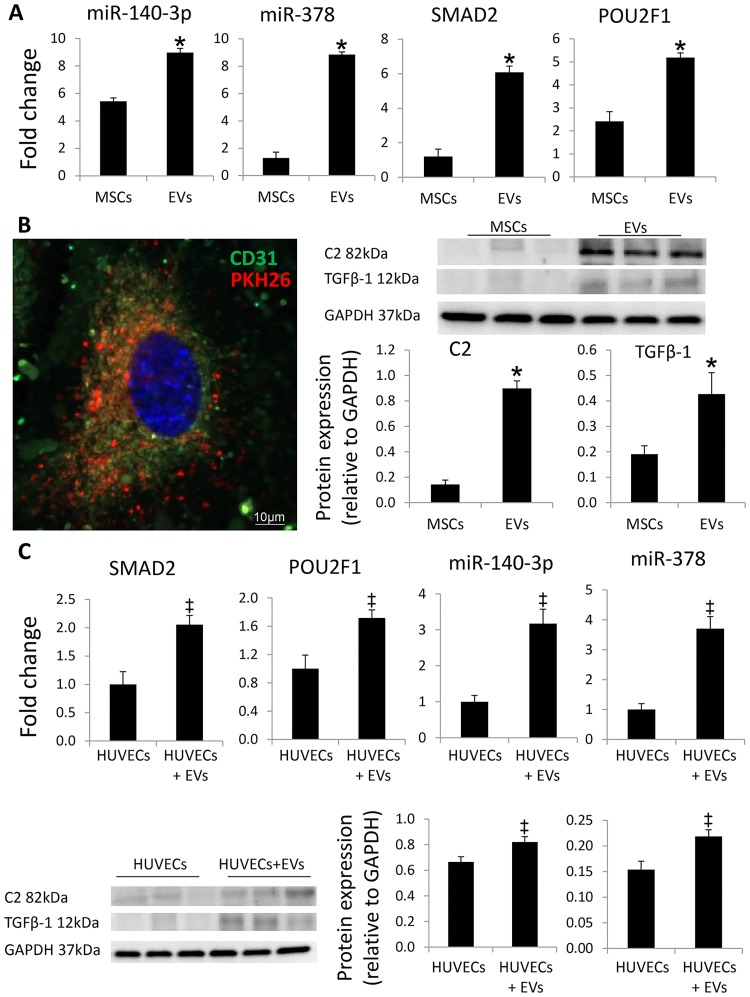
Validation of RNAseq and proteomic analysis. Expression of the candidate miRNAs (miR-140-3p and miR-378), genes (SMAD2 and POU2F1), and proteins (C2 and TGFβ-1) was concordant with the RNAseq and proteomics findings. MSC-derived EVs (PKH26, red) were internalized by cultured of human umbilical vein endothelial cells (HUVECs) (CD31, green). DAPI DAPI = blue, nuclei. Incubation of HUVECs with EVs increased HUVEC expression of miR-140-3p, miR-378, SMAD2, POU2F1, C2, and TGFβ-1. *p<0.05 vs. MSCs, ‡p<0.05 vs. HUVECs.

Expression of the candidate miRNAs, genes, and proteins followed the same patterns as the proteomics findings. Specifically, miR-140-3p, miR-378, SMAD2, POU2F1, C2, and TGFβ-1 were higher in EVs compared to their parent MSCs (Fig 6A).

### HUVEC studies

MSC-derived EVs were internalized by cultured HUVECs (Fig 6B). Incubation of HUVECs with EVs increased HUVEC expression of miR-140-3p, miR-378, SMAD2, POU2F1, C2, and TGFβ-1.

### Integrated miRNA, mRNA, and protein analysis

**Fig 7:**
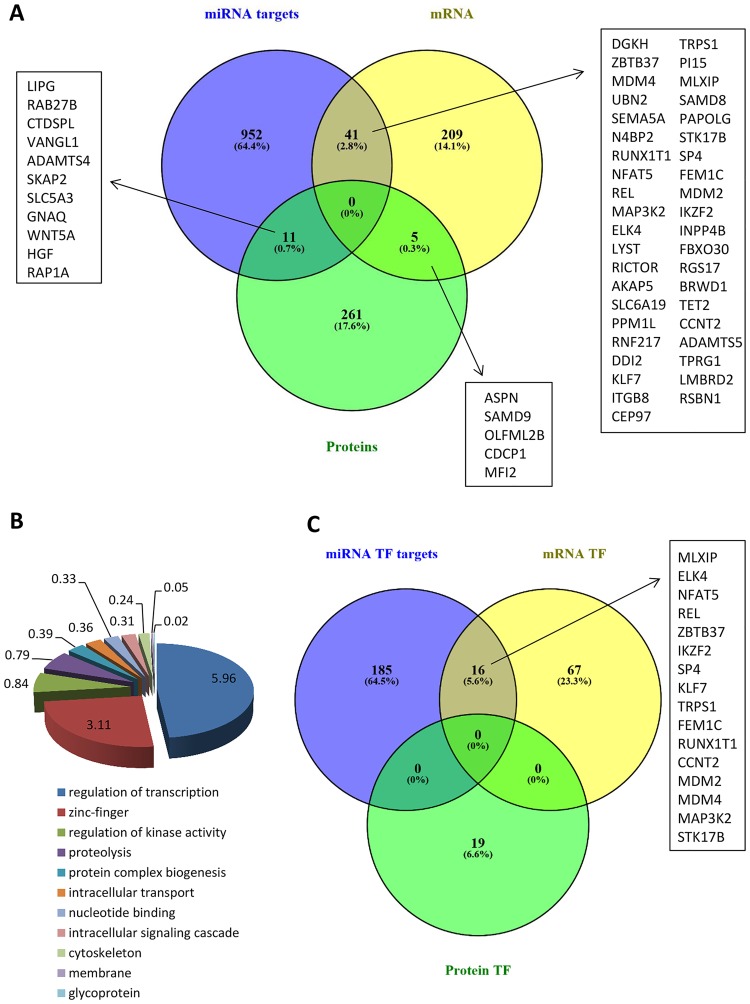
Interactions among miRNA, mRNA and proteins enriched in EVs. A. Venn diagram showing distribution of miRNA, mRNA, and proteins enriched in EVs, and their interactions. B. Functional annotation clustering of 41 common miRNA targets and mRNAs enriched in EVs. C. Distribution of transcription factor (TF) miRNA targets, TF mRNA, and TF proteins enriched in EVs.

Venn diagrams identified 41 EV-enriched mRNAs that also are targeted by miRNAs enriched in EVs (Fig 7A). Functional annotation analysis revealed that these mRNAs primarily encode proteins involved in regulation of transcription, including specifically the subcategory of zinc finger proteins (Fig 7B). However, the collection of EV-enriched proteins does not overlap with predicted miRNA targets (n = 11, 0.7%) or mRNAs (n = 5, 0.3%). The few genes that appear in common are likely attributable to stochastic events. A Venn diagram of TF mRNAs, miRNA that target TFs, and TF proteins enriched in EVs revealed that 16 TF mRNAs may represent direct miRNA targets (Fig 7C, S1 Table), yet none of the TF proteins provided are related to either TF mRNAs or TFs targeted by miRNAs. Hence, the EVs do not appear to contain mRNAs and their encoded protein, but rather one or the other.

**Fig 8:**
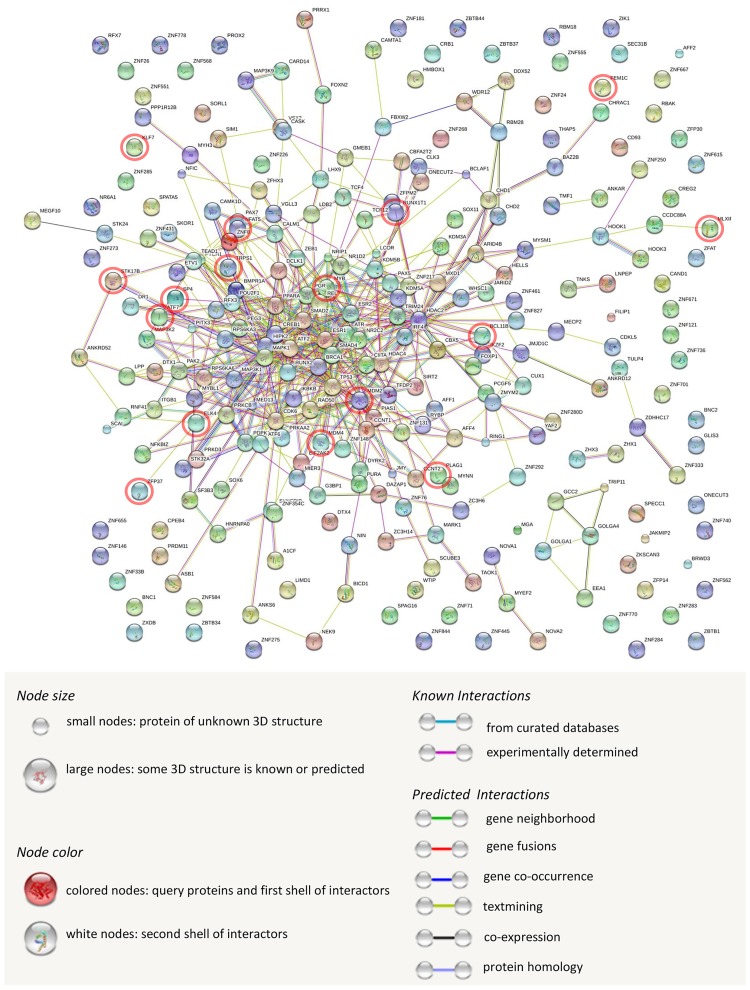
Interactions between Transcription Factor (TF) miRNA targets and TF mRNAs enriched in EVs. TF miRNA targets and mRNA TF network derived from STRING. Nodes represent TF miRNA targets and mRNA TFs, and color lines their interactions according to the functional association networks from the STRING database. Red circles indicate common (TF) miRNA targets and TF mRNAs enriched in EVs.

To examine the regulatory interactions between mRNAs that encoded TFs and miRNAs that target these TF mRNAs, we used STRING as a computational method to infer gene functional interaction networks. We found a total of 529 known or predicted interactions and the most relevant of these include protein homology, co-expression, and experimentally determined interactions (S2 Table, Fig 8). Several TF mRNAs and TF-related miRNA targets exhibited high interaction scores, including MDM2-MDM4 (0.99), RUNX1T1- CBFA2T2 (0.95), CCNT2-CCNT1 (0.91), MAP3K2-PAK2 (0.91), and ELK1-MAPK-1 (0.85). Thus, mRNAs and miRNAs enriched in EVs may be linked to the same regulatory circuits.

The data generated in this study have been deposited in the NCBI Gene Expression Omnibus (GEO, accession number GSE87790), the Mass spectrometry Interactive Virtual Environment (MassIVE, accession number MSV000080245), and ProteomeXchange (accession number PXD005147).

## Discussion

The beneficial effects of MSCs have been attributed in part to the release of EVs, which participate in intercellular communication between MSCs and damaged cells. Coupled with their potential to alter the phenotype of recipient cells and exert tissue trophic and reparative effects [29], EVs have emerged as a novel and viable alternative to whole cell therapies. In this study, we have evaluated the molecular relatedness and putative functions of mRNAs, miRNAs, and proteins packed in MSC-derived EVs. Importantly, we found that the mRNA, miRNA, and protein content of EVs is distinct and mostly independent. Proteins present in EVs are distinct from those encoded by mRNAs included in EVs, and most of the miRNAs enriched in EVs target mRNAs distinct from those enriched in EVs. Hence, there is no direct regulatory correlation between mRNAs, miRNAs and proteins, in the sense that EVs do not incorporate mRNAs that encode for the same proteins that they contain. These findings are consistent with the premise that mRNAs are not translated in EVs and miRNAs do not actively silence translation of their cognate mRNAs.

A key advantage of the molecular diversity of mRNAs, miRNAs, and proteins packed in EVs is that EVs can provide recipient cells with several different regulatory options: (i) EV proteins can directly exert a biochemical effect upon release from EVs, (ii) mRNAs can be translated to generate proteins at levels greater than could be achieved by shuttling them within EVs (or that would otherwise be incompatible with EV generation or transport), and (iii) miRNAs can suppress translation of proteins and/or degrade mRNA species in cells targeted by EVs. Collectively, our observations suggest that EVs shuttle a selective three-component cargo (mRNA, miRNA and protein), which in molecular proportions might be different from that of donor cells. This cargo selectivity is consistent with the important putative roles of EVs as vectors of MSC function and intercellular communication, as well as the potential role of MSC-derived EVs in stimulating tissue repair.

Because EVs are enriched with an independent molecular tool kit comprising distinct proteins, mRNAs, and miRNAs, it is essential to link their activity with regulatory principles and biological activities that can be leveraged for therapeutic gain. Our study shows that the mRNAs and miRNAs of MSC-derived EVs are linked to pathways that control transcription in the nucleus, presumably to regulate gene expression in recipient cells. In contrast, proteins present in EVs seem to be associate with a broad spectrum of cellular signal transduction pathways that may control how recipient cells respond to external signals. Thus, our integrated analysis of the RNA and protein cargo of EVs from MSCs reveals an apparent dichotomy in both the cellular location and specific biochemical mechanisms that are targeted. This dichotomy would permit a model in which EVs command both the regulatory input and phenotypic expression output of the cells that they interact with.

Moreover, the identity of specific miRNAs, mRNAs, and proteins provides important clues about their regulatory potential. For example, our miRNA-seq analysis revealed that miR-183, miR-378, miR-140-3p, and miR-222 are enriched in EVs compared to MSCs. These miRNAs modulate differentiation commitments and fate of MSCs. For example, miR-183 regulates β1 integrin expression and thus cell adhesion of MSCs [30], whereas miR-378 promotes MSC survival and vascularization under hypoxic-ischemic conditions [31]. miR-140 (both miR-140-5p and miR-140-3p) regulates osteogenic lineage commitment in undifferentiated MSCs [32], whereas miR-222 regulates the expression of cell cycle regulatory proteins, endothelial cell function, and angiogenesis [33]. Therefore, transfer of these miRNAs to other damaged MSCs might promote proliferation and neovascularization, and their transfer to other cell types might condition them to interact with MSCs or EVs.

Functional annotation clustering analysis of mRNAs targeted by this set of miRNAs identified genes primarily associated to transcription. Likewise, EVs were selectively enriched with 255 genes encoding positive or negative regulators of transcription, including TRPS1, ELK4, KLF7, and NRIP1. Contrarily, functional classification of 277 proteins enriched in EVs revealed an important diversity of biological roles, including glycoproteins, extracellular matrix, blood coagulation, inflammatory response, TGF-β signaling pathway, and angiogenic proteins. Importantly, our RNAseq and proteomic findings correlate with previous observations in human MSCs [34] [35], suggesting that the MSC-derived EV ‘regulome’ has a high homology with the human counterpart. Hence, MSC-derived EVs contain an extensive and heterogeneous genetic and protein cargo capable of modulating several pathways in recipient cells. Indeed, we have recently shown in pigs with metabolic syndrome and renovascular disease that 4 weeks after intra-renal injection MSC-derived EVs incorporated into several cell types in the post-stenotic kidney, including proximal tubules, distal tubules, and macrophages [36], and attenuated tubular injury, fibrosis, and inflammation. In the current study, we noticed that MSC-derived EVs were enriched with genes, miRNAs, and proteins capable of modulating renal injury and fibrosis (e.g. TGFBI, TGFβ1, MMP-2), and inflammation (e.g. TNFAIP6, NFKBIZ, miR-140-3p), suggesting that the MSC-derived EV cargo matches the need of the tissue.

Furthermore, to test the capability of EVs to transfer their cargo to target cells, we incubated HUVECs with EVs. We found that MSC-derived EVs were internalized by cultured HUVECs. Notably, incubation of HUVECs with EVs increased the expression of several miRNAs, genes, and proteins enriched in those EVs, suggesting transfer of genetic and protein cargo.

Interestingly, despite the mostly independent EV miRNA, mRNA, and protein content, we identified a significant number of EV-enriched mRNAs that did overlap with miRNA target genes upregulated in EVs. miRNAs are small non-coding RNA molecules that act as post-transcriptional negative regulators through base-pairing interactions with their targets mRNAs, leading to their degradation or translational repression. Among overlapping EV-enriched mRNAs and miRNA target genes are Serine/Threonine Kinase-17b (STK17B) and Tet Methylcytosine Dioxygenase-2 (TET2), which modulate MSC proliferation [37, 38], and RPTOR Independent Companion Of MTOR Complex-2 (RICTOR), an adaptor protein of the mammalian target of rapamycin (mTOR) multiprotein complex-2 that modulates MSC differentiation [39]. Therefore, EV miRNA-induced posttranscriptional regulation of these genes may be one of the mechanisms by which MSCs regulate their neighbors’ proliferation and differentiation.

Alternatively, miRNAs can inhibit cellular transcriptional repressors, and, thereby allow transcription. For example, Zinc Finger and BTB Domain Containing-32 (ZBTB32), a common mRNA and miRNA target gene enriched in EVs, functions as a transcriptional repressor to regulate the differentiation and activation of helper T-cells [40]. Therefore, miRNA-induced transcriptional regulation of this gene may be implicated in MSC-induced modulation of the immune response. Likewise, runt-related transcription factor-1 (RUNX1T1), a member of the myeloid translocation gene family that recruits a range of corepressors to facilitate transcriptional repression, overlapped between EV-enriched mRNAs and miRNA target genes. Importantly, down-regulation of this gene is required for adipocyte differentiation of MSCs [41]. Therefore, post-transcriptional regulation of RUNX1T1 expression may trigger adipocyte differentiation in EV recipient cells.

Functional annotation analysis of common miRNA target genes and mRNAs enriched in EVs showed that over 70% are related to TFs. These proteins bind to specific DNA sequences, controlling transcription of genetic information from DNA to mRNAs. MSC self-renewal, proliferation, potency, and fate are regulated by coordinated transcription factor networks [42]. In line with this notion, gene functional interaction network analysis revealed multiple associations with overlapping mRNAs and miRNA target TFs, including interactions between MDM2 and MDM4, negative regulators of p53 that play a key role in the initiation of the MSC adipogenic program [43]. Furthermore, we found interactions of MAPK1 with SMAD2 and SMAD4, which might be implicated in regulation of both cell survival and apoptosis [44]. Therefore, our observations suggest that interactions between EV TFs participate in the transcriptional control of cellular function in recipient cells.

We acknowledge limitations to this study, including the low number of samples and inability to identify MSC and EV post-translational changes by LC-MS/MS proteomic analysis. Despite these caveats, this study has a number of strengths, including the novel comprehensive characterization of the mRNA, miRNA, and proteomic cargo of porcine MSC-derived EVs, and a joint analysis using an integrated system level approach to elucidate their molecular networks and interactions.

## Conclusions

In summary, our study shows that porcine MSC-derived EVs contain mRNAs, miRNAs, and proteins capable of modifying recipient cell phenotype and function, modulating multiple cellular pathways, and activating regenerative mechanisms. Moreover, differences in miRNA, mRNA, and protein composition between EVs and their parent MSCs suggest a complex mechanism of EV cargo sequestration and packaging. Notably, we identified a significant number of overlapping mRNAs and miRNAs TFs enriched in EVs, suggesting that interactions between mRNA and miRNA target TFs may be an important mechanism driving MSC-based repair. These observations support proposed shuttling mechanisms mediated by EV-dependent signaling between MSCs and recipient cells, and encourage development of EV-based regenerative strategies. Our present studies provide a platform for further studies to elucidate the molecular mechanisms by which proteins, transcriptional factors, and translational regulators transferred by MSC-derived EVs may activate tissue repair in recipient cells.

## Supporting information

S1 Table Transcription factors enriched in Extracellular Vesicles (EVs). Sixteen transcription factors enriched in EVs that overlap with mRNA transcription factor targets of 4 miRNAs enriched in EVs.
(PDF)

S2 Table Interactions between mRNA Transcription Factors (TFs). Interactions between mRNA TFs and miRNA TF target genes enriched in extracellular vesicles (EVs) using Search Tool for the Retrieval of Interacting Genes (STRING).
(PDF)
