# Supplementary material for: Integrated transcriptomic and proteomic analysis of the molecular cargo of extracellular vesicles derived from porcine adipose tissue-derived mesenchymal stem cells
Source: PLoS One. 2017 Mar 23;12(3):e0174303. doi: 10.1371/journal.pone.0174303 (PMC5363917; doi:10.1371/journal.pone.0174303)
Supplement: S1 Table — Sixteen transcription factors enriched in EVs that overlap with mRNA transcription factor targets of 4 miRNAs enriched in EVs. (PDF) [file pone.0174303.s001.pdf]

Table S1. Sixteen transcription factors enriched in extracellular vesicles (EVs) that overlap with mRNA transcription factor targets of 4 miRNAs enriched in EVs.

**Official gene symbol**	**Gene name**
MLXIP	MLX Interacting Protein
ELK4	ETS Transcription Factor
NFAT5	Nuclear Factor Of Activated T-Cells 5, Tonicity-Responsive
REL	REL Proto-Oncogene, NF-KB Subunit
ZBTB37	Zinc Finger And BTB Domain Containing 37
IKZF2	IKAROS Family Zinc Finger 2
SP4	Sp4 Transcription Factor
KLF7	Kruppel-Like Factor 7
TRPS1	Transcriptional Repressor GATA Binding 1
FEM1C	Fem-1 Homolog C
RUNX1T1	RUNX1 Translocation Partner 1
CCNT2	Cyclin T2
MDM2	MDM2 Proto-Oncogene
MDM4	MDM4, P53 Regulator
MAP3K2	Mitogen-Activated Protein Kinase Kinase Kinase 2
STK17B	Serine/Threonine Kinase 17b
