# Supplementary material for: Integrated transcriptomic and proteomic analysis of the molecular cargo of extracellular vesicles derived from porcine adipose tissue-derived mesenchymal stem cells
Source: PLoS One. 2017 Mar 23;12(3):e0174303. doi: 10.1371/journal.pone.0174303 (PMC5363917; doi:10.1371/journal.pone.0174303)
Supplement: S2 Table — Interactions between mRNA TFs and miRNA TF target genes enriched in extracellular vesicles (EVs) using Search Tool for the Retrieval of Interacting Genes (STRING). (PDF) [file pone.0174303.s002.pdf]

Table S2. Interactions between mRNA transcription factors (TFs) and miRNA TF target genes enriched in extracellular vesicles (EVs) using Search Tool for the Retrieval of Interacting Genes (STRING).

Node 1	Node 2	Neighborhood on chromosome	Gene fusion	Phylogenetic co-occurrence	Homology	Co-expression	Experimentally determined interaction	Database annotated	Automated textmining	Combined score
AFF1	CCNT1	0	0	0	0	0	0.958	0	0.351	0.971
AFF1	AFF4	0	0	0	0.667	0	0.68	0	0.72	0.755
AFF1	PAX5	0	0	0	0	0	0.566	0	0.27	0.669
AFF1	CDK6	0	0	0	0	0	0	0	0.48	0.48
AFF4	CCNT1	0	0	0	0	0	0.985	0	0.899	0.998
AFF4	CCNT2	0	0	0	0	0	0.302	0	0.33	0.512
ANKRD52	CDK6	0	0	0	0	0	0.149	0	0.37	0.44
ANKRD52	STK17B	0	0	0	0	0	0.074	0	0.417	0.437
ANKS6	CDK6	0	0	0	0	0	0.149	0	0.37	0.44
ANKS6	NEK9	0	0	0	0	0	0	0	0.402	0.402
ARID4B	HELLS	0	0	0	0	0	0.101	0	0.375	0.414
ATF2	MAPK1	0	0	0	0	0	0.861	0.9	0.567	0.993
ATF2	ESR1	0	0	0	0	0	0.074	0.9	0.328	0.932
ATF2	BMPR1A	0	0	0	0	0	0	0.9	0.073	0.903
ATF6	TP53	0	0	0	0	0	0	0	0.458	0.457
ATF6	EIF2AK2	0	0	0	0	0	0	0	0.436	0.436
ATF6	ATF2	0	0	0	0	0	0	0	0.412	0.412
ATF7	MAPK1	0	0	0	0	0	0.299	0	0.481	0.62
ATF7	ATF2	0	0	0	0.947	0	0.574	0	0.642	0.588
ATR	TP53	0	0	0	0	0	0.998	0.9	0.583	0.999
ATR	RAD50	0	0	0	0	0.113	0.796	0	0.827	0.966
ATR	DCLK1	0	0	0	0	0	0.587	0	0.561	0.81
ATR	CDK6	0	0	0	0	0.065	0.357	0	0.45	0.64
ATR	SIRT2	0	0	0	0	0	0.234	0	0.292	0.434
BAZ2B	CHRAC1	0	0	0	0	0	0.111	0	0.399	0.443
BAZ2B	CHD1	0	0	0	0	0.077	0.202	0	0.25	0.4
BCL11B	CBX5	0	0	0	0	0	0.808	0	0.117	0.823
BCL11B	FOXP1	0	0	0	0	0	0.102	0	0.504	0.536
BCL11B	MYB	0	0	0	0	0	0.087	0	0.41	0.438
BCLAF1	CHD1	0	0	0	0	0	0.57	0	0	0.57
BCLAF1	CLK3	0	0	0	0	0	0.462	0	0	0.461
BCLAF1	MYB	0	0	0	0	0	0	0	0.459	0.459
BICD1	NEK9	0	0	0	0	0	0	0	0.56	0.56
BRCA1	TP53	0	0	0	0	0	0.999	0.9	0.974	0.999
BRCA1	RAD50	0	0	0	0	0	0.984	0.9	0.813	0.999

BRCA1	ATR	0	0	0	0	0	0.992	0.9	0.582	0.999
BRCA1	ESR1	0	0	0	0	0	0.999	0.9	0.815	0.999
BRCA1	POU2F1	0	0	0	0	0	0.809	0.9	0.126	0.981
BRCA1	PGR	0	0	0	0	0	0.812	0	0.81	0.962
BRCA1	ATF2	0	0	0	0	0	0	0.9	0.186	0.915
BRCA1	CREB1	0	0	0	0	0	0.812	0	0.35	0.872
BRCA1	PIAS1	0	0	0	0	0	0.808	0	0.36	0.871
BRCA1	ZNF148	0	0	0	0	0	0	0	0.813	0.813
BRCA1	SMAD4	0	0	0	0	0	0.564	0	0.501	0.773
BRCA1	SMAD2	0	0	0	0	0	0.564	0	0.342	0.7
BRCA1	LDB2	0	0	0	0	0	0.571	0	0.313	0.692
BRCA1	KDM5B	0	0	0	0	0	0.551	0	0.26	0.653
BRCA1	MDM2	0	0	0	0	0	0.235	0	0.565	0.652
BRCA1	MAPK1	0	0	0	0	0	0.576	0	0.211	0.651
BRCA1	DAZAP1	0	0	0	0	0	0.566	0	0.192	0.634
BRCA1	ATF6	0	0	0	0	0	0.576	0	0.102	0.602
BRCA1	MED13	0	0	0	0	0	0.569	0	0.112	0.6
BRCA1	PEG3	0	0	0	0	0	0.566	0	0.061	0.575
BRCA1	ELK4	0	0	0	0	0	0.566	0	0.041	0.566
BRCA1	ESR2	0	0	0	0	0	0	0	0.5	0.499
BRCA1	MDM4	0	0	0	0	0	0	0	0.5	0.499
BRCA1	MAP3K1	0	0	0	0	0	0.053	0	0.417	0.424
BRCA1	CDK6	0	0	0	0	0	0	0	0.416	0.416
BRCA1	SIRT2	0	0	0	0	0	0.276	0	0.216	0.408
BRCA1	MYB	0	0	0	0	0	0	0	0.405	0.405
CALM1	DCLK1	0	0	0	0	0	0.111	0	0.62	0.648
CALM1	ESR2	0	0	0	0	0	0.567	0	0.199	0.638
CALM1	ESR1	0	0	0	0	0	0.567	0	0.2	0.638
CALM1	MYH3	0	0	0	0	0	0.211	0	0.514	0.6
CALM1	REL	0	0	0	0	0	0.576	0	0.072	0.589
CALM1	TCF4	0	0	0	0	0	0.529	0	0.054	0.535
CALM1	STK17B	0	0	0	0	0	0.111	0	0.395	0.439
CAMK1D	CALM1	0	0	0	0	0	0.111	0.8	0.682	0.938
CAMK1D	MAPK1	0	0	0.206	0.671	0	0.25	0	0.489	0.401
CASK	CARD14	0	0	0	0	0	0	0	0.747	0.748
CASK	CALM1	0	0	0	0	0	0	0	0.506	0.506
CASK	GMEB1	0	0	0	0	0	0.455	0	0	0.455
CCDC88A	MLXIP	0	0	0	0	0	0	0	0.504	0.504
CCNT1	ESR1	0	0	0	0	0	0.576	0	0.826	0.923
CCNT2	CCNT1	0	0	0	0.894	0	0	0.9	0.79	0.908
CDK6	CCNT1	0	0	0	0	0	0.298	0	0.439	0.589
CDK6	CCNT2	0	0	0	0	0	0.298	0	0.364	0.534
CDK6	ESR1	0	0	0	0	0	0	0	0.508	0.508
CDK6	ASB1	0	0	0	0	0	0.149	0	0.37	0.44

CHD1	ARID4B	0	0	0	0	0.071	0.213	0	0.51	0.61
CHD1	RBM28	0	0	0	0	0.281	0	0	0.205	0.404
CHD2	ARID4B	0	0	0	0	0	0.213	0	0.51	0.597
CHD2	RBM28	0	0	0	0	0.281	0	0	0.2	0.4
CIITA	CCNT1	0	0	0	0	0	0	0	0.897	0.897
CIITA	MAPK1	0	0	0	0	0	0.564	0	0.533	0.787
CIITA	SMAD2	0	0	0	0	0	0	0	0.707	0.707
CIITA	HDAC4	0	0	0	0	0	0.575	0	0.105	0.603
CLK3	TP53	0	0	0	0	0	0.462	0	0.047	0.465
CREB1	TP53	0	0	0	0	0	0.972	0	0.938	0.998
CREB1	RPS6KA3	0	0	0	0	0	0.566	0.9	0.977	0.998
CREB1	MAPK1	0	0	0	0	0	0	0.9	0.949	0.994
CREB1	CIITA	0	0	0	0	0	0	0.8	0.944	0.988
CREB1	ATF2	0	0	0	0	0	0.083	0.9	0.884	0.988
CREB1	RPS6KA6	0	0	0	0	0	0	0.9	0.668	0.965
CREB1	MAP3K1	0	0	0	0	0	0	0.9	0.185	0.915
CREB1	ATF7	0	0	0	0	0	0.659	0	0.727	0.903
CREB1	PRKCB	0	0	0	0	0	0	0.8	0.179	0.828
CREB1	HIPK2	0	0	0	0	0	0	0	0.821	0.821
CREB1	SMAD2	0	0	0	0	0	0	0	0.775	0.776
CREB1	REL	0	0	0	0	0	0	0	0.776	0.776
CREB1	MYB	0	0	0	0	0	0	0	0.766	0.766
CREB1	DR1	0	0	0	0	0.067	0.644	0	0.304	0.749
CREB1	POU2F1	0	0	0	0	0	0.576	0	0.347	0.711
CREB1	ESR1	0	0	0	0	0	0	0	0.666	0.666
CREB1	SMAD4	0	0	0	0	0	0	0	0.622	0.622
CREB1	ATR	0	0	0	0	0	0.566	0	0.156	0.618
CREB1	PPARA	0	0	0	0	0	0	0	0.61	0.61
CREB1	ATF6	0	0	0	0	0	0.198	0	0.505	0.586
CREB1	PTCH1	0	0	0	0	0	0	0	0.583	0.583
CREB1	PGR	0	0	0	0	0	0	0	0.536	0.536
CREB1	STK32A	0	0	0	0	0	0	0	0.534	0.534
CREB1	CAMK1D	0	0	0	0	0	0.105	0	0.492	0.526
CREB1	PRKAA2	0	0	0	0	0	0	0	0.496	0.496
CREB1	CDK6	0	0	0	0	0	0	0	0.489	0.489
CREB1	DCLK1	0	0	0	0	0	0	0	0.477	0.477
CREB1	PDPK1	0	0	0	0	0	0	0	0.444	0.444
CREB1	RFX3	0	0	0	0	0	0.385	0	0.133	0.444
CREB1	RUNX2	0	0	0	0	0	0	0	0.429	0.429
CREB1	PIAS1	0	0	0	0	0	0.272	0	0.248	0.429
CREB1	LHX9	0	0	0	0	0	0	0	0.405	0.405
CUX1	BCL11B	0	0	0	0	0	0.082	0	0.611	0.628
CUX1	FOXP1	0	0	0	0	0	0	0	0.412	0.412
DCLK1	MAPK1	0	0	0	0.682	0.091	0.25	0	0.593	0.419

DDX52	RBM28	0	0	0	0	0.841	0	0	0.349	0.892
DDX52	WDR12	0	0	0	0	0.89	0	0	0.046	0.89
DR1	PPARA	0	0	0	0	0	0	0	0.661	0.661
DYRK2	TP53	0	0	0	0	0	0	0.9	0.85	0.984
ELK4	MAPK1	0	0	0	0	0	0.102	0.8	0.276	0.858
ELK4	RNF41	0	0	0	0	0	0	0	0.549	0.549
ESR2	ESR1	0	0	0	0.935	0	0.988	0.9	0.981	0.998
ESR2	MAPK1	0	0	0	0	0	0.601	0.8	0.259	0.935
ESR2	PGR	0	0	0	0.72	0	0	0.9	0.813	0.922
ESR2	PPARA	0	0	0	0.642	0	0	0.9	0.417	0.914
ESR2	NR1D2	0	0	0	0.633	0	0	0.9	0.362	0.912
ESR2	NR2C2	0	0	0	0.64	0	0	0.9	0.129	0.903
ESR2	TRIM24	0	0	0	0	0	0.793	0	0.094	0.805
ESR2	NRIP1	0	0	0	0	0	0.546	0	0.393	0.713
ESR2	SMAD4	0	0	0	0	0	0.567	0	0.195	0.636
ESR2	SMAD2	0	0	0	0	0	0.567	0	0.138	0.61
ESR2	TP53	0	0	0	0	0	0	0	0.58	0.58
ESR2	MYB	0	0	0	0	0	0	0	0.455	0.455
ETV1	RPS6KA3	0	0	0	0	0	0	0	0.824	0.824
ETV1	PPARA	0	0	0	0	0	0	0	0.8	0.8
ETV1	PAX7	0	0	0	0	0	0	0	0.468	0.468
FBXW2	MYB	0	0	0	0	0	0.573	0	0	0.573
FBXW2	WDR12	0	0	0.44	0	0	0	0	0	0.44
FBXW2	CAMTA1	0	0	0	0	0	0	0	0.411	0.411
FOXN2	PRRX1	0	0	0	0	0	0.086	0	0.373	0.402
G3BP1	PDPK1	0	0	0	0	0	0.92	0	0	0.92
G3BP1	TP53	0	0	0	0	0	0.564	0	0.18	0.627
G3BP1	DAZAP1	0	0	0	0	0.423	0	0	0.132	0.478
GCC2	TRIP11	0	0	0	0	0.076	0	0	0.506	0.524
GMEB1	CBFA2T2	0	0	0	0	0	0	0	0.486	0.485
GOLGA1	GCC2	0	0	0	0	0	0	0	0.725	0.725
GOLGA1	GOLGA4	0	0	0	0	0	0	0	0.725	0.725
GOLGA1	EEA1	0	0	0	0	0	0	0	0.478	0.478
GOLGA4	GCC2	0	0	0	0	0.063	0	0	0.771	0.776
GOLGA4	TRIP11	0	0	0	0	0.114	0	0	0.628	0.656
GOLGA4	EEA1	0	0	0	0	0.128	0	0	0.361	0.418
HDAC2	TP53	0	0	0	0	0	0.999	0	0.582	0.999
HDAC2	ARID4B	0	0	0	0	0	0.95	0.9	0.9	0.999
HDAC2	ZNF217	0	0	0	0	0	0.93	0.9	0.437	0.995
HDAC2	MXD1	0	0	0	0	0	0.849	0.9	0.138	0.985
HDAC2	BCL11B	0	0	0	0	0	0.931	0	0.389	0.956
HDAC2	ZMYM2	0	0	0	0	0	0.932	0	0.342	0.953
HDAC2	SMAD2	0	0	0	0	0	0.304	0.9	0.335	0.949
HDAC2	MDM2	0	0	0	0	0	0.422	0.9	0.197	0.949

HDAC2	SMAD4	0	0	0	0	0	0.249	0.9	0.349	0.946
HDAC2	CREB1	0	0	0	0	0	0.812	0	0.714	0.944
HDAC2	CIITA	0	0	0	0	0	0.917	0	0.341	0.943
HDAC2	BRCA1	0	0	0	0	0	0.847	0	0.398	0.904
HDAC2	PCGF5	0	0	0	0	0	0	0.9	0.064	0.902
HDAC2	HELLS	0	0	0	0	0.1	0.832	0	0.389	0.9
HDAC2	HDAC4	0	0	0	0.657	0	0.458	0.72	0.928	0.891
HDAC2	WHSC1	0.063	0	0	0	0	0.843	0	0.286	0.886
HDAC2	CHD1	0	0	0	0	0.078	0.302	0	0.798	0.859
HDAC2	KDM5B	0	0	0	0	0	0.653	0	0.513	0.824
HDAC2	NRIP1	0	0	0	0	0	0.566	0.36	0.325	0.796
HDAC2	CHD2	0	0	0	0	0.078	0.302	0	0.699	0.789
HDAC2	ESR1	0	0	0	0	0	0.574	0	0.5	0.777
HDAC2	ATR	0	0	0	0	0	0.662	0	0.344	0.769
HDAC2	SIRT2	0	0	0	0	0	0.276	0	0.694	0.769
HDAC2	TRIM24	0	0	0	0	0	0.653	0	0.348	0.764
HDAC2	KDM5A	0	0	0	0	0	0.475	0	0.529	0.743
HDAC2	ZFPM2	0	0	0	0	0	0.618	0	0.236	0.696
HDAC2	ZNF827	0	0	0	0	0	0.619	0	0.181	0.674
HDAC2	RUNX1T1	0	0	0	0	0	0.573	0	0.244	0.663
HDAC2	CBX5	0	0	0	0	0.096	0	0.54	0.253	0.662
HDAC2	MECP2	0	0	0	0	0	0	0	0.63	0.63
HDAC2	IKZF2	0	0	0	0	0	0.557	0	0.181	0.621
HDAC2	PIAS1	0	0	0	0	0.072	0.317	0	0.403	0.589
HDAC2	MAPK1	0	0	0	0	0	0.149	0	0.449	0.511
HDAC2	KDM3A	0	0	0	0	0	0.276	0	0.289	0.463
HDAC2	MYB	0	0	0	0	0	0.101	0	0.424	0.46
HDAC2	ATF2	0	0	0	0	0	0.149	0	0.351	0.424
HDAC2	MIER3	0	0	0	0	0	0.413	0	0	0.413
HDAC4	ESR1	0	0	0	0	0	0.812	0.9	0.281	0.985
HDAC4	CBX5	0	0	0	0	0	0.57	0.54	0.071	0.8
HDAC4	SIRT2	0	0	0	0	0	0.276	0	0.649	0.735
HDAC4	ATF2	0	0	0	0	0	0.623	0	0.178	0.677
HDAC4	MAPK1	0	0	0	0	0	0.6	0	0.184	0.66
HIPK2	TP53	0	0	0	0	0	0.998	0.9	0.952	0.999
HIPK2	MYB	0	0	0	0	0	0.805	0	0.888	0.977
HIPK2	SMAD2	0	0	0	0	0	0.571	0	0.718	0.874
HIPK2	MAP3K1	0	0	0	0.609	0	0.667	0	0.16	0.684
HIPK2	PTCH1	0	0	0	0	0	0.571	0	0.047	0.573
HIPK2	ZNF217	0	0	0	0	0.096	0.102	0	0.4	0.47
HIPK2	MDM4	0	0	0	0	0	0	0	0.436	0.436
HNRNPA0	SF3B3	0	0	0	0	0	0	0.9	0.092	0.905
HOOK1	HOOK3	0	0	0	0.959	0	0.556	0.9	0.862	0.955
HOOK1	CCDC88A	0	0	0	0	0	0	0	0.609	0.609

HOOK1	ZNF250	0	0	0	0	0	0.434	0	0	0.434
IKBKB	PRKCB	0	0	0	0.624	0	0.919	0.9	0.257	0.992
IKBKB	MAP3K1	0	0	0	0.621	0	0.916	0.9	0.256	0.991
IKBKB	NR2C2	0	0	0	0	0	0.576	0	0.838	0.928
IKBKB	PDPK1	0	0	0	0.639	0	0	0.9	0.212	0.906
IKBKB	CCNT1	0	0	0	0	0	0	0.9	0.072	0.903
IKBKB	TP53	0	0	0	0	0	0.807	0	0.502	0.899
IKBKB	REL	0	0	0	0	0	0	0	0.722	0.722
IKBKB	EIF2AK2	0	0	0	0.631	0	0.564	0	0.068	0.568
IKBKB	CALM1	0	0	0	0	0	0.352	0	0.197	0.457
IKZF2	HDAC4	0	0	0	0	0	0.603	0	0.199	0.668
IRF4	CIITA	0	0	0	0	0	0	0.9	0.472	0.944
IRF4	ATF2	0	0	0	0	0	0	0.9	0.047	0.9
IRF4	REL	0	0	0	0	0	0	0	0.897	0.897
IRF4	PAX5	0	0	0	0	0	0	0	0.722	0.722
IRF4	FOXP1	0	0	0	0	0	0.1	0	0.47	0.502
JMJD1C	ESR2	0	0	0	0	0	0.484	0	0	0.483
JMJD1C	ANKRD12	0	0	0	0	0.078	0	0	0.432	0.454
JMJD1C	ZMYM2	0	0	0	0	0	0	0	0.409	0.41
JMY	TP53	0	0	0	0	0	0.576	0	0.389	0.729
KDM3A	PPARA	0	0	0	0	0	0	0	0.609	0.609
KDM5A	RUNX2	0	0	0	0	0	0	0	0.821	0.821
KDM5A	PGR	0	0	0	0	0	0	0	0.81	0.81
KDM5A	ESR1	0	0	0	0	0	0.57	0	0.126	0.608
KDM5A	MXD1	0	0	0	0	0	0.576	0	0	0.576
KDM5A	NR2C2	0	0	0	0	0	0.57	0	0.043	0.57
KDM5A	ARID4B	0	0	0	0	0	0.087	0	0.533	0.555
KDM5A	KDM3A	0	0	0	0	0	0	0	0.547	0.547
KDM5B	HDAC4	0	0	0	0	0	0.666	0	0.219	0.728
KDM5B	ZNF217	0	0	0	0	0	0.569	0	0.3	0.685
KDM5B	KDM3A	0	0	0	0	0	0	0	0.613	0.613
KDM5B	ONECUT2	0	0	0	0	0.101	0.443	0	0.057	0.486
LCOR	ESR1	0	0	0	0	0	0.57	0.9	0.346	0.969
LCOR	NRIP1	0	0	0	0	0	0	0	0.646	0.646
LCOR	HDAC4	0	0	0	0	0	0.57	0	0.126	0.608
LHX9	LDB2	0	0	0	0	0	0.878	0	0.856	0.981
LHX9	VSX2	0	0	0	0	0	0	0	0.777	0.777
LHX9	PTCH1	0	0	0	0	0	0	0	0.459	0.459
LHX9	FOXN2	0	0	0	0	0	0.086	0	0.373	0.402
LPP	MAPK1	0	0	0	0	0	0	0	0.616	0.616
LPP	RPS6KA6	0	0	0	0	0	0	0	0.47	0.47
MAP3K1	MAPK1	0	0	0	0.665	0	0.945	0	0.692	0.958
MAP3K1	PAK2	0	0	0	0.697	0	0.362	0.9	0.271	0.938
MAP3K1	SMAD2	0	0	0	0	0	0	0.9	0.186	0.915

MAP3K1	ESR1	0	0	0	0	0	0.567	0	0.348	0.705
MAP3K1	TP53	0	0	0	0	0	0.576	0	0.33	0.703
MAP3K1	MAP3K2	0	0	0	0.729	0	0.583	0	0.64	0.653
MAP3K1	DTX1	0	0	0	0	0	0.576	0	0	0.576
MAP3K1	PRKCB	0	0	0	0.628	0	0.397	0	0.771	0.568
MAP3K1	MIER3	0	0	0	0	0	0	0	0.502	0.502
MAP3K2	PAK2	0	0	0	0.704	0	0.447	0.8	0.687	0.907
MAP3K2	MAPK1	0	0	0	0.675	0	0.327	0	0.752	0.489
MAP3K9	CARD14	0.462	0	0	0	0	0.077	0	0.166	0.549
MAP3K9	CASK	0.462	0	0	0.615	0	0.077	0	0.166	0.508
MAPK1	ESR1	0	0	0	0	0	0.919	0.9	0.344	0.994
MARK1	HDAC4	0	0	0	0	0	0.331	0	0.617	0.733
MARK1	TAOK1	0	0	0	0.624	0	0.576	0	0.583	0.666
MARK1	ZC3H14	0	0	0	0	0	0.071	0	0.653	0.663
MDM2	TP53	0	0	0	0	0.167	0.999	0.9	0.981	0.999
MDM2	MDM4	0	0	0	0.786	0	0.999	0.9	0.856	0.999
MDM2	ESR1	0	0	0	0	0	0.962	0	0.472	0.979
MDM2	PIAS1	0	0	0	0	0	0.564	0.9	0.163	0.96
MDM2	HIPK2	0	0	0	0	0	0.912	0	0.5	0.954
MDM2	PDPK1	0	0	0	0	0	0	0.9	0.243	0.921
MDM2	ATR	0	0	0	0	0	0	0.9	0.16	0.912
MDM2	RAD50	0	0	0	0	0	0.665	0	0.286	0.75
MDM2	JMY	0	0	0	0	0	0.576	0	0.359	0.716
MDM2	DYRK2	0	0	0	0	0	0.566	0	0.242	0.656
MDM2	ESR2	0	0	0	0	0	0.576	0	0.21	0.65
MDM2	CDK6	0	0	0	0	0	0	0	0.502	0.502
MDM4	TP53	0	0	0	0	0	0.999	0.8	0.949	0.999
MDM4	SMAD4	0	0	0	0	0	0.564	0	0.824	0.92
MECP2	CDKL5	0	0	0	0	0	0	0	0.704	0.704
MED13	PPARA	0	0	0	0	0	0	0.9	0.103	0.906
MED13	ESR1	0	0	0	0	0	0.571	0	0.101	0.597
MYB	CDK6	0	0	0	0	0	0.15	0.9	0.95	0.995
MYB	TP53	0	0	0	0	0	0	0.9	0.937	0.993
MYB	ESR1	0	0	0	0	0	0	0	0.912	0.912
MYB	NRIP1	0	0	0	0	0	0	0	0.846	0.846
MYB	PIAS1	0	0	0	0	0.074	0.576	0	0.315	0.707
MYB	MAPK1	0	0	0	0	0	0.603	0	0.263	0.695
MYB	ZFHX3	0	0	0	0	0	0.576	0	0.249	0.668
MYB	ATF2	0	0	0	0	0	0.11	0	0.55	0.582
MYB	REL	0	0	0	0	0	0	0	0.518	0.518
MYB	SF3B3	0	0	0	0	0.077	0.448	0	0	0.468
MYB	RPS6KA6	0	0	0	0	0	0	0	0.468	0.468
MYB	ATF7	0	0	0	0	0	0.11	0	0.389	0.433
MYB	PGR	0	0	0	0	0	0	0	0.407	0.407

MYBL1	PITX3	0	0	0	0	0	0.575	0	0.737	0.883
MYBL1	CDK6	0	0	0	0	0	0.15	0	0.641	0.682
MYBL1	HIPK2	0	0	0	0	0	0.571	0	0.243	0.661
MYBL1	CREB1	0	0	0	0	0	0	0	0.525	0.525
MYBL1	RFX3	0	0	0	0	0	0.245	0	0.328	0.471
MYBL1	RPS6KA6	0	0	0	0	0	0	0	0.468	0.468
MYBL1	RPS6KA3	0	0	0	0	0	0	0	0.468	0.468
MYBL1	SF3B3	0	0	0	0	0.077	0.448	0	0	0.468
MYBL1	ATF2	0	0	0	0	0	0.11	0	0.356	0.402
MYBL1	ATF7	0	0	0	0	0	0.11	0	0.356	0.402
MYEF2	NOVA2	0	0	0	0	0	0.2	0	0.704	0.753
MYSM1	HELLS	0	0	0	0	0.109	0.724	0	0.73	0.928
MYSM1	ARID4B	0	0	0	0	0	0.363	0	0.584	0.723
MYSM1	MYB	0	0	0	0	0	0.078	0	0.477	0.497
MYSM1	HOOK1	0	0	0	0	0	0	0	0.479	0.479
NFAT5	REL	0	0	0	0	0	0	0	0.601	0.601
NFAT5	CREB1	0	0	0	0	0	0	0	0.569	0.569
NFAT5	TP53	0	0	0	0	0	0	0	0.402	0.402
NFIC	ZFHX3	0	0	0	0	0	0	0	0.455	0.455
NFIC	POU2F1	0	0	0	0	0	0	0	0.407	0.407
NFKBIZ	CDK6	0	0	0	0	0	0.149	0	0.425	0.49
NFKBIZ	REL	0	0	0	0	0	0.149	0	0.424	0.488
NIN	BICD1	0	0	0	0	0.072	0.575	0	0.12	0.622
NIN	PIAS1	0	0	0	0	0	0.576	0	0	0.576
NOVA1	MYEF2	0	0	0	0	0	0.2	0	0.704	0.753
NR1D2	ESR1	0	0	0	0.632	0	0	0.9	0.277	0.909
NR1D2	PPARA	0	0	0	0.693	0	0	0.9	0.291	0.908
NR2C2	ESR1	0	0	0	0.635	0	0.812	0.9	0.198	0.981
NR2C2	PPARA	0	0	0	0.655	0	0	0.9	0.225	0.906
NR2C2	NR1D2	0	0	0	0.639	0	0	0.9	0.196	0.905
NR2C2	HDAC4	0	0	0	0	0	0.575	0	0	0.575
NRIP1	ESR1	0	0	0	0	0	0.988	0.9	0.939	0.999
NRIP1	PPARA	0	0	0	0	0	0.808	0.9	0.58	0.991
NRIP1	MAPK1	0	0	0	0	0	0	0.9	0.052	0.901
NRIP1	ZNF217	0	0	0	0	0	0	0.36	0.482	0.654
NRIP1	PGR	0	0	0	0	0	0	0	0.566	0.566
NRIP1	GMEB1	0	0	0	0	0	0	0	0.537	0.537
NRIP1	HDAC4	0	0	0	0	0	0	0.36	0.138	0.424
PAK2	ITGB1	0	0	0	0	0.064	0	0.9	0.1	0.908
PAK2	SMAD2	0	0	0	0	0.079	0	0	0.872	0.877
PAK2	MAPK1	0	0	0.379	0.679	0	0.691	0	0.389	0.758
PAK2	CDK6	0	0	0.329	0.72	0	0.297	0	0.372	0.418
PAX5	MYB	0	0	0	0	0	0.576	0.9	0.902	0.995
PAX5	TP53	0	0	0	0	0	0	0	0.896	0.896

PAX5	TCF12	0	0	0	0	0	0	0	0.836	0.836
PAX5	SOX11	0	0	0	0	0	0	0	0.834	0.834
PAX5	TCF4	0	0	0	0	0	0	0	0.652	0.653
PAX5	REL	0	0	0	0	0	0	0	0.495	0.495
PAX5	CIITA	0	0	0	0	0	0	0	0.472	0.472
PAX7	MYB	0	0	0	0	0	0	0	0.821	0.821
PAX7	PTCH1	0	0	0	0	0	0	0	0.783	0.783
PAX7	SIM1	0	0	0	0	0	0	0	0.436	0.436
PAX7	PEG3	0	0	0	0	0	0	0	0.434	0.434
PDPK1	PRKCB	0	0	0	0.723	0	0.717	0.9	0.662	0.975
PDPK1	RPS6KA6	0	0	0	0.727	0	0.537	0.9	0.791	0.962
PDPK1	MAPK1	0	0	0	0.671	0	0.11	0.9	0.393	0.918
PDPK1	TP53	0	0	0	0	0	0	0	0.5	0.499
PEG3	TP53	0	0	0	0	0	0	0	0.863	0.863
PGR	ESR1	0	0	0	0.711	0	0.812	0.9	0.943	0.985
PGR	MAPK1	0	0	0	0	0	0.601	0.9	0.216	0.966
PGR	PPARA	0	0	0	0.63	0	0	0.9	0.506	0.917
PGR	NR1D2	0	0	0	0.621	0	0	0.9	0.144	0.904
PGR	NR2C2	0	0	0	0.627	0	0	0.9	0.071	0.901
PGR	TP53	0	0	0	0	0	0	0	0.814	0.814
PIAS1	ESR1	0	0	0	0	0	0.566	0	0.325	0.694
PITX3	MYB	0	0	0	0	0	0.575	0	0.738	0.884
PITX3	CDK6	0	0	0	0	0	0.149	0	0.591	0.638
PITX3	SMAD2	0	0	0	0	0	0	0	0.418	0.418
PLAG1	PIAS1	0	0	0	0	0	0.585	0	0.047	0.588
POU2F1	ATF2	0	0	0	0	0	0.109	0.9	0.159	0.918
POU2F1	ESR1	0	0	0	0	0	0.572	0	0.264	0.671
POU2F1	PGR	0	0	0	0	0	0.572	0	0.243	0.662
POU2F1	TEAD1	0	0	0	0	0	0.566	0	0.14	0.61
POU2F1	SP4	0	0	0	0	0	0.543	0	0	0.543
POU2F1	ZFHX3	0	0	0	0	0	0	0	0.415	0.415
PPARA	MAPK1	0	0	0	0	0	0.601	0.9	0.317	0.97
PPARA	ESR1	0	0	0	0.65	0	0	0.9	0.7	0.924
PPARA	SIRT2	0	0	0	0	0	0	0	0.406	0.406
PPP1R12B	MYH3	0	0	0	0	0	0.109	0	0.488	0.524
PRKAA2	PPARA	0	0	0	0	0	0	0	0.859	0.859
PRKAA2	TP53	0	0	0	0	0	0.108	0	0.834	0.846
PRKAA2	HDAC4	0	0	0	0	0	0.331	0	0.412	0.59
PRKAA2	MAPK1	0	0	0.23	0.657	0	0.3	0	0.269	0.4
PRKCB	MAPK1	0	0	0	0.641	0	0.347	0.8	0.681	0.896
PRKCB	ATF2	0	0	0	0	0	0.149	0.8	0.128	0.838
PRKCB	ITGB1	0	0	0	0	0	0	0.8	0.114	0.815
PRKCB	PRKD3	0	0	0	0.629	0	0	0.8	0.191	0.811
PTCH1	ZNF431	0	0	0	0	0	0.088	0	0.829	0.838

PTCH1	TP53	0	0	0	0	0	0	0	0.586	0.586
PTCH1	BMPR1A	0	0	0	0	0	0.169	0	0.376	0.459
PTCH1	CDK6	0	0	0	0	0	0.254	0	0.273	0.434
PURA	CCNT1	0	0	0	0	0	0.576	0	0.32	0.699
PURA	EIF2AK2	0	0	0	0	0	0.461	0	0.076	0.48
RAD50	DCLK1	0	0	0	0	0	0.301	0	0.367	0.538
RAD50	SIRT2	0.089	0	0	0	0	0.149	0	0.29	0.401
REL	TP53	0	0	0	0	0	0	0	0.929	0.93
REL	ESR1	0	0	0	0	0	0	0	0.507	0.507
REL	ATF2	0	0	0	0	0	0	0	0.426	0.426
REL	PPARA	0	0	0	0	0	0	0	0.415	0.414
RFX3	ATR	0	0	0	0	0	0.757	0	0.134	0.78
RFX3	DCLK1	0	0	0	0	0	0.319	0	0.545	0.677
RFX3	DTX1	0	0	0	0	0	0	0	0.483	0.483
RFX3	MYB	0	0	0	0	0	0.245	0	0.33	0.473
RFX3	HDAC4	0	0	0	0	0	0	0	0.405	0.405
RING1	PCGF5	0	0	0	0	0	0.334	0	0.46	0.625
RNF41	ANKRD52	0	0	0	0	0	0	0	0.434	0.434
RPS6KA3	MAPK1	0	0	0	0.697	0	0.995	0.9	0.703	0.999
RPS6KA3	PDPK1	0	0	0	0.73	0	0.936	0.9	0.974	0.995
RPS6KA3	RPS6KA6	0	0	0	0.983	0	0	0.9	0.564	0.9
RPS6KA3	ESR1	0	0	0	0	0	0.807	0	0.419	0.883
RPS6KA3	MYB	0	0	0	0	0	0	0	0.589	0.59
RPS6KA3	TP53	0	0	0	0	0	0	0	0.509	0.509
RPS6KA3	LPP	0	0	0	0	0	0	0	0.489	0.49
RPS6KA3	ATF2	0	0	0	0	0	0.149	0	0.335	0.41
RPS6KA6	MAPK1	0	0	0	0.699	0	0.27	0.9	0.569	0.936
RUNX1T1	CBFA2T2	0	0	0	0.968	0	0.944	0	0.949	0.945
RUNX1T1	TCF12	0	0	0	0	0	0.574	0	0.181	0.636
RUNX1T1	TCF4	0	0	0	0	0	0.574	0	0.127	0.612
RUNX1T1	GMEB1	0	0	0	0	0	0	0	0.486	0.485
RUNX2	SMAD4	0	0	0	0	0	0	0.9	0.909	0.99
RUNX2	SMAD2	0	0	0	0	0	0.575	0	0.957	0.981
RUNX2	HDAC4	0	0	0	0	0	0.92	0	0.474	0.956
RUNX2	MAPK1	0	0	0	0	0	0	0.9	0.182	0.914
RUNX2	ATF6	0	0	0	0	0	0	0	0.822	0.822
RUNX2	ZNF354C	0	0	0	0	0	0	0	0.8	0.8
RUNX2	TP53	0	0	0	0	0	0.576	0	0.507	0.782
RUNX2	BMPR1A	0	0	0	0	0	0.569	0	0.423	0.741
RUNX2	PPARA	0	0	0	0	0	0.08	0	0.587	0.603
RUNX2	SOX6	0	0	0	0	0	0	0	0.589	0.589
RUNX2	ESR1	0	0	0	0	0	0.08	0	0.507	0.527
RUNX2	PAX5	0	0	0	0	0	0.301	0	0.313	0.499
RUNX2	ITGB1	0	0	0	0	0	0	0	0.466	0.465

RUNX2	PTCH1	0	0	0	0	0	0	0	0.457	0.457
RYBP	RING1	0	0	0	0	0	0.643	0	0.875	0.953
RYBP	TP53	0	0	0	0	0	0.566	0	0.165	0.622
RYBP	MDM2	0	0	0	0	0	0.566	0	0.052	0.57
RYBP	PCGF5	0	0	0	0	0	0.302	0	0.223	0.434
SCAI	ITGB1	0	0	0	0	0	0	0.9	0.07	0.903
SIM1	VSX2	0	0	0	0	0	0	0	0.447	0.447
SKOR1	SMAD4	0	0	0	0	0	0	0	0.716	0.716
SKOR1	SMAD2	0	0	0	0	0	0	0	0.501	0.502
SMAD2	MAPK1	0	0	0	0	0	0.576	0.9	0.653	0.984
SMAD2	BMPR1A	0	0	0	0	0	0.248	0.8	0.868	0.978
SMAD2	ESR1	0	0	0	0	0	0.804	0	0.416	0.88
SMAD2	ZNF8	0	0	0	0	0	0.566	0	0.067	0.577
SMAD4	SMAD2	0	0	0	0.75	0	0.999	0.9	0.995	0.999
SMAD4	ATF2	0	0	0	0	0	0.576	0.9	0.878	0.994
SMAD4	BMPR1A	0	0	0	0	0	0.341	0.9	0.871	0.99
SMAD4	ESR1	0	0	0	0	0	0.567	0.9	0.474	0.975
SMAD4	MAPK1	0	0	0	0	0	0.576	0.8	0.49	0.953
SMAD4	TP53	0	0	0	0	0	0	0	0.943	0.943
SMAD4	PIAS1	0	0	0	0	0	0.686	0	0.797	0.934
SMAD4	PPARA	0	0	0	0	0	0	0	0.863	0.863
SMAD4	ZNF8	0	0	0	0	0	0.566	0	0.064	0.576
SMAD4	CDK6	0	0	0	0	0	0	0	0.412	0.412
STK17B	MAPK1	0	0	0.206	0.663	0	0.25	0	0.489	0.404
STK24	TEAD1	0	0	0	0	0	0	0	0.474	0.474
STK24	MEGF10	0	0	0	0	0.41	0	0	0	0.41
STK32A	PDPK1	0	0	0.272	0.744	0	0.453	0	0.669	0.572
STK32A	MAPK1	0	0	0.256	0.661	0.07	0.27	0	0.357	0.418
SYNCRIP	A1CF	0	0	0	0.854	0	0.812	0	0.524	0.826
SYNCRIP	TP53	0	0	0	0	0	0	0	0.824	0.824
SYNCRIP	HNRNPA0	0	0	0	0.651	0	0.57	0	0.25	0.602
TCF12	SMAD2	0	0	0	0	0	0.569	0	0.067	0.58
TEAD1	PPARA	0	0	0	0	0	0	0.9	0.058	0.901
TEAD1	MAPK1	0	0	0	0	0	0.299	0	0.588	0.698
TEAD1	MAP3K2	0	0	0	0	0	0.149	0	0.424	0.488
TFDP2	SMAD4	0	0	0	0	0	0	0.9	0.218	0.918
TFDP2	BRCA1	0	0	0	0	0	0	0.9	0.063	0.902
TFDP2	SMAD2	0	0	0	0	0	0	0.9	0.047	0.9
TFDP2	RYBP	0	0	0	0	0	0	0.9	0	0.9
TFDP2	CBX5	0	0	0	0	0	0	0.9	0.045	0.9
TFDP2	CDK6	0	0	0	0	0.064	0.102	0	0.519	0.56
TFDP2	MYNN	0	0	0	0	0	0	0	0.424	0.424
TFDP2	MYB	0	0	0	0	0.097	0.269	0	0.181	0.412
TMF1	ANKAR	0	0	0	0	0	0	0	0.86	0.86

TNKS	LNPEP	0	0	0	0	0	0.803	0	0.311	0.858
TP53	PIAS1	0	0	0	0	0	0.998	0	0.458	0.999
TP53	MAPK1	0	0	0	0	0	0.988	0.8	0.508	0.998
TP53	SMAD2	0	0	0	0	0	0.987	0	0.912	0.998
TP53	EIF2AK2	0	0	0	0	0	0.808	0.9	0.825	0.996
TP53	ESR1	0	0	0	0	0	0.812	0	0.856	0.971
TP53	RAD50	0	0	0	0	0	0	0.9	0.666	0.965
TP53	SIRT2	0	0	0	0	0	0.276	0	0.912	0.934
TP53	CDK6	0	0	0	0	0	0	0	0.894	0.894
TP53	PPARA	0	0	0	0	0	0	0	0.606	0.606
TP53	HDAC4	0	0	0	0	0	0.233	0	0.415	0.532
TP53	ATF2	0	0	0	0	0	0	0	0.472	0.472
TRIM24	ESR1	0	0	0	0	0	0.997	0	0.408	0.998
TRIM24	TP53	0	0	0	0	0	0.912	0	0.31	0.936
TRIM24	CBX5	0	0	0	0	0	0.805	0	0.325	0.863
TRIM24	PGR	0	0	0	0	0	0.573	0	0.266	0.673
TRIM24	NRIP1	0	0	0	0	0	0	0	0.581	0.581
TRIM24	MYB	0	0	0	0	0	0.225	0	0.37	0.491
TRPS1	RUNX2	0	0	0	0	0	0	0	0.877	0.878
VGLL3	TEAD1	0	0	0	0	0	0	0	0.86	0.86
VGLL3	KDM5B	0	0	0	0	0	0.443	0	0	0.443
WDR12	RBM28	0	0	0	0	0.825	0.945	0	0.161	0.991
WHSC1	HDAC4	0.063	0	0	0	0	0.22	0	0.288	0.434
WHSC1	KDM5B	0	0	0	0	0	0.201	0	0.302	0.418
WHSC1	IRF4	0	0	0	0	0	0	0	0.405	0.405
YAF2	RING1	0	0	0	0	0	0.105	0	0.774	0.789
YAF2	PCGF5	0	0	0	0	0	0.302	0	0.38	0.548
ZC3H14	DAZAP1	0	0	0	0	0	0.722	0	0	0.722
ZC3H6	ZNF292	0	0	0	0	0	0	0	0.718	0.718
ZC3H6	DAZAP1	0	0	0	0	0	0.383	0	0.378	0.599
ZDHHC17	ZNF333	0	0	0	0	0.079	0.374	0	0.072	0.418
ZDHHC17	TULP4	0	0	0	0	0	0	0	0.418	0.418
ZEB1	SMAD4	0	0	0	0	0	0	0	0.896	0.896
ZEB1	MYB	0	0	0	0	0	0	0	0.876	0.876
ZEB1	SMAD2	0	0	0	0	0	0.576	0	0.535	0.794
ZEB1	ZFHX3	0	0	0	0	0	0	0	0.624	0.624
ZEB1	TP53	0	0	0	0	0	0	0	0.568	0.568
ZEB1	CDK6	0	0	0	0	0	0.292	0	0.31	0.49
ZEB1	ESR1	0	0	0	0	0	0	0	0.469	0.469
ZFP37	ELK4	0	0	0	0	0	0	0	0.412	0.412
ZFPM2	LHX9	0	0	0	0	0	0	0	0.553	0.553
ZHX3	ZHX1	0	0	0	0.854	0	0.809	0	0.862	0.833
ZMYM2	CUX1	0	0	0	0	0	0	0.9	0.175	0.913
ZMYM2	ZNF280D	0	0	0	0	0	0.226	0	0.758	0.805

ZMYM2	AFF4	0	0	0	0	0	0	0	0.68	0.68
ZMYM2	ZNF292	0	0	0	0	0	0	0	0.444	0.444
ZNF131	ESR1	0	0	0	0	0	0.59	0	0.13	0.628
ZNF148	TP53	0	0	0	0	0	0.576	0	0.855	0.936
ZNF148	HDAC4	0	0	0	0	0	0.604	0	0.26	0.694
ZNF217	HDAC4	0	0	0	0	0	0.105	0.72	0.199	0.781
ZNF217	CBX5	0	0	0	0	0	0	0.54	0	0.54
ZNF461	HDAC2	0	0	0	0	0	0.623	0	0.427	0.775
